# Modular network inference between miRNA–mRNA expression profiles using weighted co-expression network analysis

**DOI:** 10.1515/jib-2021-0029

**Published:** 2021-11-22

**Authors:** Nisar Wani, Debmalya Barh, Khalid Raza

**Affiliations:** Computer Science and Engineering Department, Govt. College of Engineering and Technology Safapora, Ganderbal Kashmir, J&K, India; Institute of Integrative Omics and Applied Biotechnology (IIOAB), Nonakuri, Purba Medinipur, WB, India; Department of Genetics, Ecology and Evolution, Institute of Biological Sciences, Federal University of Minas Gerais, Belo Horizonte, Minas Gerais, Brazil; Department of Computer Science, Jamia Millia Islamia, New Delhi, India

**Keywords:** gene expression, hubs, miRNA, module detection, module eigengene networks, network inference

## Abstract

Connecting transcriptional and post-transcriptional regulatory networks solves an important puzzle in the elucidation of gene regulatory mechanisms. To decipher the complexity of these connections, we build co-expression network modules for mRNA as well as miRNA expression profiles of breast cancer data. We construct gene and miRNA co-expression modules using the weighted gene co-expression network analysis (WGCNA) method and establish the significance of these modules (Genes/miRNAs) for cancer phenotype. This work also infers an interaction network between the genes of the turquoise module from mRNA expression data and hubs of the turquoise module from miRNA expression data. A pathway enrichment analysis using a miRsystem web tool for miRNA hubs and some of their targets, reveal their enrichment in several important pathways associated with the progression of cancer.

## 1 Introduction

Gene expression programs encode the instructions for organism development, its physiological functions, and morphological characteristics. These programs are regulated at multiple levels and involve interaction between different regulatory factors. The first level of interaction occurs at the transcription level wherein DNA binding proteins called transcription factors (TFs) attach themselves to the promoter sequences near the transcription start sites and help build transcription initiation complex. These TFs also bind themselves to regulatory sequences, such as enhancers, and can control gene expression either by activating or repressing transcription of related genes [1–3]. The way these gene expression regulators interact with their targets can be described in the form of a transcriptional regulatory network (TRN). In a TRN, the genes and TFs serve as network nodes and the edges represent the direct interactions between them. TRNs are highly complex due to the complex connectivity patterns of their nodes and the number of regulatory links present in the network.

Building models of TRNs provides a systems approach to study important functions of the living organisms that control and co-ordinate its development and physiology of all vital organs. A thorough understanding of TRNs is a vital tool in the hands of research scientists and can help in solving problems in the research domains ranging from basic to applied bioinformatics.

Disease mechanisms that arise due to the dysfunction of TRNs can also be explained by understanding underlying regulatory processes. TRNs also guide the design of efficient strategies to select novel drug targets and to understand cellular engineering. A range of computational methods for the inference of TRNs has been proposed over a couple of decades. These methods operate on either a single data source (transcriptome) or a combination of multiple data sources (i.e., genome, transcriptome, methylome, and epigenome, etc.) that play an essential role in gene expression regulation. A comprehensive review of these methods can be found in [4, 5].

Another level of gene expression regulation takes place after the process of transcription is over. This post-transcriptional gene regulation is primarily mediated through small non-coding RNAs usually referred to as miRNAs. These miRNA are approximately 22 nt in length and have become a major attraction in gene regulation studies for a variety of disease mechanisms [6]. The discovery of miRNA’s has expanded the scope of gene regulation studies [7, 8], and extensive literature has been published since then to understand the functions of miRNAs and their role in post-transcriptional regulation. They are found in almost all eukaryotes, including humans [9]. They regulate up to 30% of the protein-coding genes in humans and account for nearly 1–5% of the total genome. They are reported to have a ubiquitous role in post-transcriptional gene regulation [10, 11] by targeting the 3′ untranslated region (UTR) of the mRNA, thereby resulting in either degradation of the target mRNA or its translational repression [12]. Since miRNAs target translational templates of proteins, they perform key functions in many biological processes, such as cell proliferation, cell differentiation, cell growth, development, and apoptosis. However, any alterations to their functioning have been related to various pathological disorders, including tumorigenesis [9]. According to miRNA target databases, miRNAs and mRNAs can establish different regulatory relationships. These relationships can be either one-to-many, where a single miRNA can regulate many target genes or many-to-one, where one gene may be the target of multiple miRNAs. Further studies delving into the miRNAs and mRNAs interactions have reported even many-to-many relationships between these genomic entities using computational analysis [9, 13, 14].

To investigate how miRNAs and genes jointly affect complex human diseases, we adopt the network approach proposed by Zhang and Horvath [15], wherein weighted co-expression network modules are built from miRNA and mRNA expression datasets to uncover the nature of complex relationships between genes and their regulators among miRNAs. Since individual genes do not act in isolation but interact with each other and other genomic entities and jointly affect human health. Therefore, inferring and analyzing networks of gene regulation (i.e., transcriptional and post-transcriptional) provides an important framework to search for genes/regulators that play an essential role in complex biological functions and diseases. Further, the integration of miRNAs co-expression modules can provide regulatory links between transcriptional and post-transcriptional regulatory mechanisms.

## 2 Related literature

Various studies have been conducted based on integrated miRNA and mRNA expression analysis mainly on different types of cancers [16–21]. These studies incorporated large scale datasets of mRNA and miRNA expression profiles to infer regulatory interactions between miRNAs and their targets genes [18–21]. For example, a bi-clique approach proposed by Peng et al. [18] uses the expression profile of miRNA and mRNA datasets for module construction. However, the modules obtained in this way resulted in very few miRNAs being included in each module, thereby, making it difficult to explain the complexities of relationships between miRNAs and their targets. Another important study aimed at constructing miRNA–mRNA regulatory modules uses bi-clustering together with a Gaussian Bayesian Network to infer miRNA–gene relationships [21]. Seo et al. [22] combined correlation and bi-clustering approaches to construct miRNA–mRNA modules to identify and rank miRNAs that are related to cancer. The ranking is done based on influence scores derived from significant correlations between miRNA–mRNA pairs. the integration of both expression profiles resulted in the identification of a large number of miRNAs related to cancer and helps in understanding their regulatory mechanisms.

A recent study by Pham et al. [23] adopted a different approach to infer miRNA–mRNA interaction networks from breast cancer data. They proposed an invariant causal prediction (ICP) method based on the causal inference approach. The ICP method searches for the miRNA–mRNA pairs wherein causal relationships exist across different cancer subtypes. The predicted miRNA–mRNA regulatory relationships are then validated by transfection data using the miRLAB R package [24] and other experimentally validated databases. A similar study that infers miRNA–mRNA regulatory networks employs Funrich [25], an analysis tool used for the prediction of TF targets of miRNAs. The study also uses a miRNet database for target gene prediction. Glioblastoma multiforme (GBM) mRNA and miRNA expression datasets downloaded from the TCGA database were subjected to differential expression analysis. Subsequently, a pathway and functional enrichment analysis on these differentially expressed gene and miRNAs was performed using Enrichr database [26]. For hub gene identification, the authors used Cytoscape software and the prognostic roles of these hubs were determined by Prognoscan and GEPIA databases. Finally, a potential miRNA–mRNA regulatory network was successfully inferred with a significant contribution to the onset and progression of GBM.

Song et al. in [27] integrated nine HCC datasets by employing rank aggregation method. These GEO datasets were subjected a weighted gene co-expression analysis revealing key modules related to hepatocellular carcinoma. A network topology analysis of the modules identified novel risk genes. The potential functions of these risk genes were further explored with the aid of miRNA–mRNA regulatory networks. Another study by Mokhtaridoost and Gönen [28] approached the same problem by devising a two-step framework to model miRNA–mRNA regulatory relationships. They formulated a regularized factor regression model (RFR) that extracts modules by decomposing the regulatory matrix into two low rank matrices, thereby grouping co-related miRNAs and mRNAs together. Another study in the domain of network medicine by Paci et al. [29], whereby the authors integrate the co-expression networks with human interactome network to predict novel putative disease genes and modules. An algorithm predicts key (switch) genes within the co-expression network which regulate disease transitions. These genes are then mapped to PPI networks to predict novel disease gene relationships. Another important study taking an integrative approach combined miRNA-seq, mRNA-seq and lncRNA datasets was carried out by Zhou et al. [30]. Here, the authors performed a WGCNA analysis and indentified key modules and hub genes as potential candidates that may contribute to the progression bovine endometritis.

Here we propose to study this relationship at the modular level between miRNA–mRNA modules by applying weighted gene co-expression network analysis (WGCNA) [15]. WGCNA is an established system biology tool to detect co-expression modules and identify key genes that serve as network hubs within these modules and associate these modules to phenotypic traits. We apply the WGCNA pipeline to both miRNA and mRNA expression profile data to construct co-expression modules and detect the hub genes in both datasets. We further explore the connectivity between miRNA and mRNA modules using the MultiMir R package [31]. MultiMir offers an interface to comprehensive databases containing predicted and experimentally validated interactions between miRNAs and their target genes in addition to their association with diseases and drugs. The identified modules from genes and miRNAs represent subgroups that are co-expressed and highly co-related and the connectivity among them derived using multiMir may further enhance our understanding of regulatory mechanisms between miRNA–mRNA regulatory interactions.

## 3 Materials and methods

### 3.1 Datasets

We downloaded miRNA and mRNA expression profiles of patients suffering from breast invasive carcinoma (BRCA) from the Broad Institute data portal (https://gdac.broadinstitute.org) that hosts TCGA (The Cancer Genome Atlas) data. For this study, we selected miRNA and mRNA expression profiles from 20 normal samples and 50 unmatched tumor samples. Besides, miRNA–gene interaction information is provided by the MultiMir R package [31]. The package provides an interface to 14 external databases that store miRNA–gene interactions, both experimentally validated and computationally predicted.

### 3.2 WGCNA pipeline

**Figure 1:**
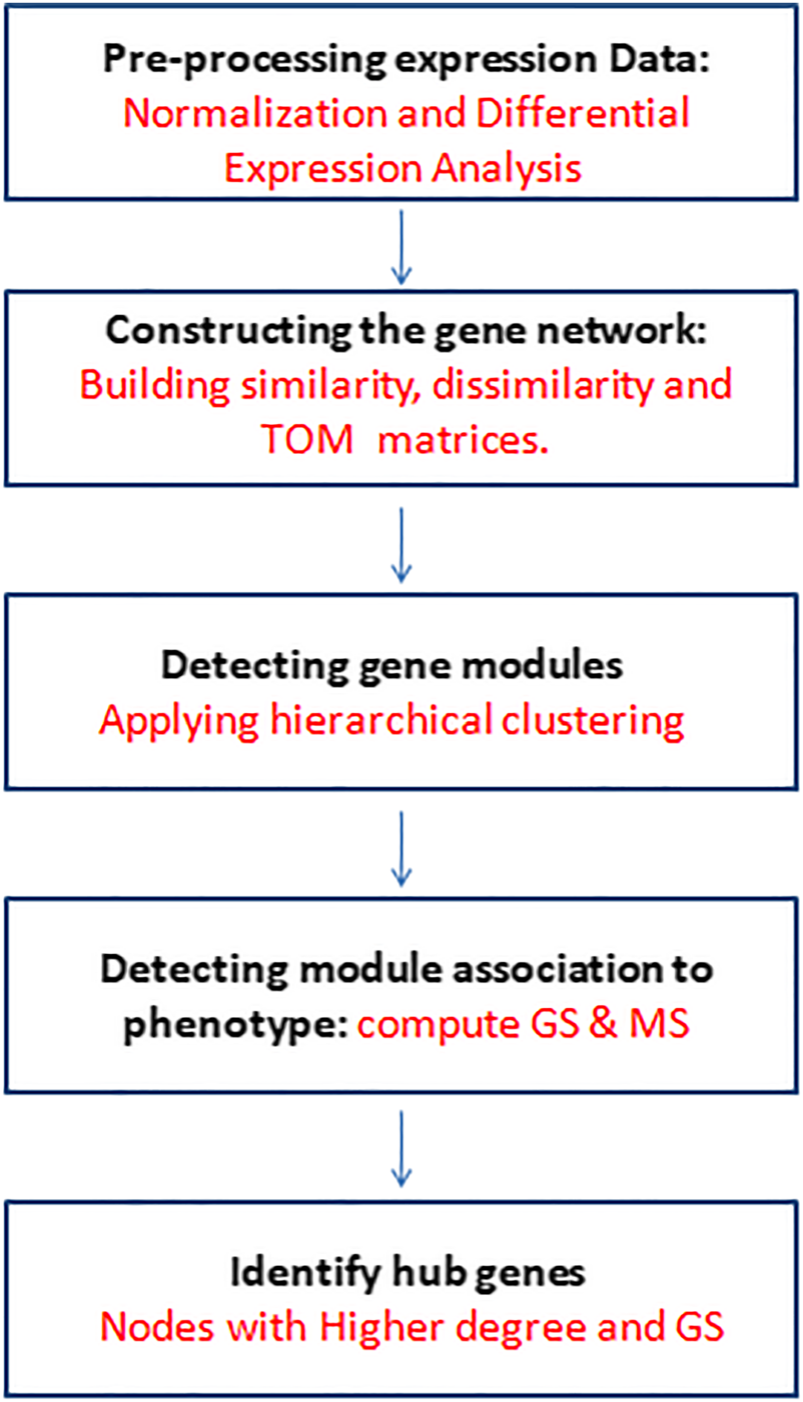
Network construction and module detection workflow.

WGCNA [15] is an important systems biology tool used to construct co-expression gene networks. Besides, WGCNA is also used to identify gene modules of gene/miRNAs and detect the role of some central players within these modules that are designated as module hubs. The WGCNA pipeline depicted in Figure 1 is as follows:

1. Pre-processing of expression data: the gene expression data are normalized using Z-scores. These normalized expression values for both miRNAs and mRNAs are subjected to differential expression analysis to obtain differentially expressed genes (DEGs) and differentially expressed miRNAs (DEMs). The limma voom package of R is used for differential expression analysis with a false discovery rate (FDR) of less than 5%.
2. Build a network of co-expressed genes formally represented by an adjacency matrix, the elements of this matrix indicate similarity in expression profiles of a gene pair.
3. Identify modules: For module identification, WGCNA employs hierarchical clustering. It first builds a similarity matrix from which it derives a dissimilarity matrix for the clustering algorithm to identify gene modules. It uses a topological overlap measure derived from the adjacency matrix. Using this measure WGCNA identifies biologically meaningful modules that can be subjected to further downstream analysis.
4. Relate modules to phenotypes: WGCNA pipeline helps associate modules to phenotypic traits. WGCNA defines a module eigengene (ME) as a representative of the expression profile of all the genes within a module. It is the first principal component that can be used to derive an association between derived modules and the phenotypic traits of interest. WGCNA defines various measures to relate genes and modules to phenotypic traits. They are (a) gene significance (GS) and (b) module significance (MS), where GS is computed by finding a correlation between genes and phenotypes of a particular module and MS is the average GS of all nodes within a given module. Modules with high MS value may represent signaling pathways characteristic of the phenotypic traits for which GS and MS have been calculated.
5. Derive inter-module associations: module eigen genes (MEs) are used as representative expression profiles for a module within the WGCNA pipeline. MEs quantify module similarities by computing the correlation between the eigengenes of different co-expression modules. Studying this relationship between modules can elucidate the similarity between gene groups in modules and their physiological functions.
6. Find key drivers in modules of interest: within the co-expression modules, there are nodes having connections to a very large number of other nodes in the network. In network terminology, nodes with higher degrees have the most and important connections within the network. In the event of these nodes malfunctioning, they affect all the connected genes within the network. WGCNA assumes that genetic networks obey the scale-free topology criterion. Doing away with the standard practice of inferring co-expression networks between gene pairs (connected = 1, unconnected = 0), the connectivity matrix is obtained by assigning weights to the edges of the connected gene pair using soft thresholding, which has been proven to yield more robust results than un-weighted networks.
7. Choosing an appropriate soft threshold parameter allows to infer co-expression networks that are nearly or close to a scale-free network. WGCNA lays strong emphasis on module trait relationships compared to relating phenotypes to individual genes, which greatly alleviates the multiple testing problem inherent in microarray data analysis.
8. WGCNA is widely applied to analysis of genomic data wherein samples are assumed to be independent of each other.

### 3.3 Network construction for module detection

For network construction, miRNA expression and mRNA expression data are normalized using Z-scores. These normalized expression values for both miRNAs and mRNAs are subjected to differential expression analysis to obtain genes and miRNAs that are differentially expressed (DEGs & DEMs) across selected tumors and normal patient samples. We use the limma voom R package for differential expression analysis with a false discovery rate set to less than 5%. The need for differential expression is to select the genes and miRNAs that show high variability across samples. The process for the construction of gene co-expression and miRNA co-expression networks follow almost similar steps. First, an expression similarity matrix is derived from expression values of both mRNA and miRNA profiles by calculating the Pearson correlation coefficient between all pairs of genes and all pairs of miRNAs. Using this similarity matrix WGCNA defines an adjacency matrix *S*_*ij*_, between the *i*th gene/miRNA and *j*th gene/miRNA.

(1) $$S_{ij}=\text{corr}\left[x_{i},x_{j}\right]$$

The co-relation values obtained from Eq. (1) are raised to a soft thresholding power of *β* > 1 to obtain weighted co-expression networks.

**Figure 2:**
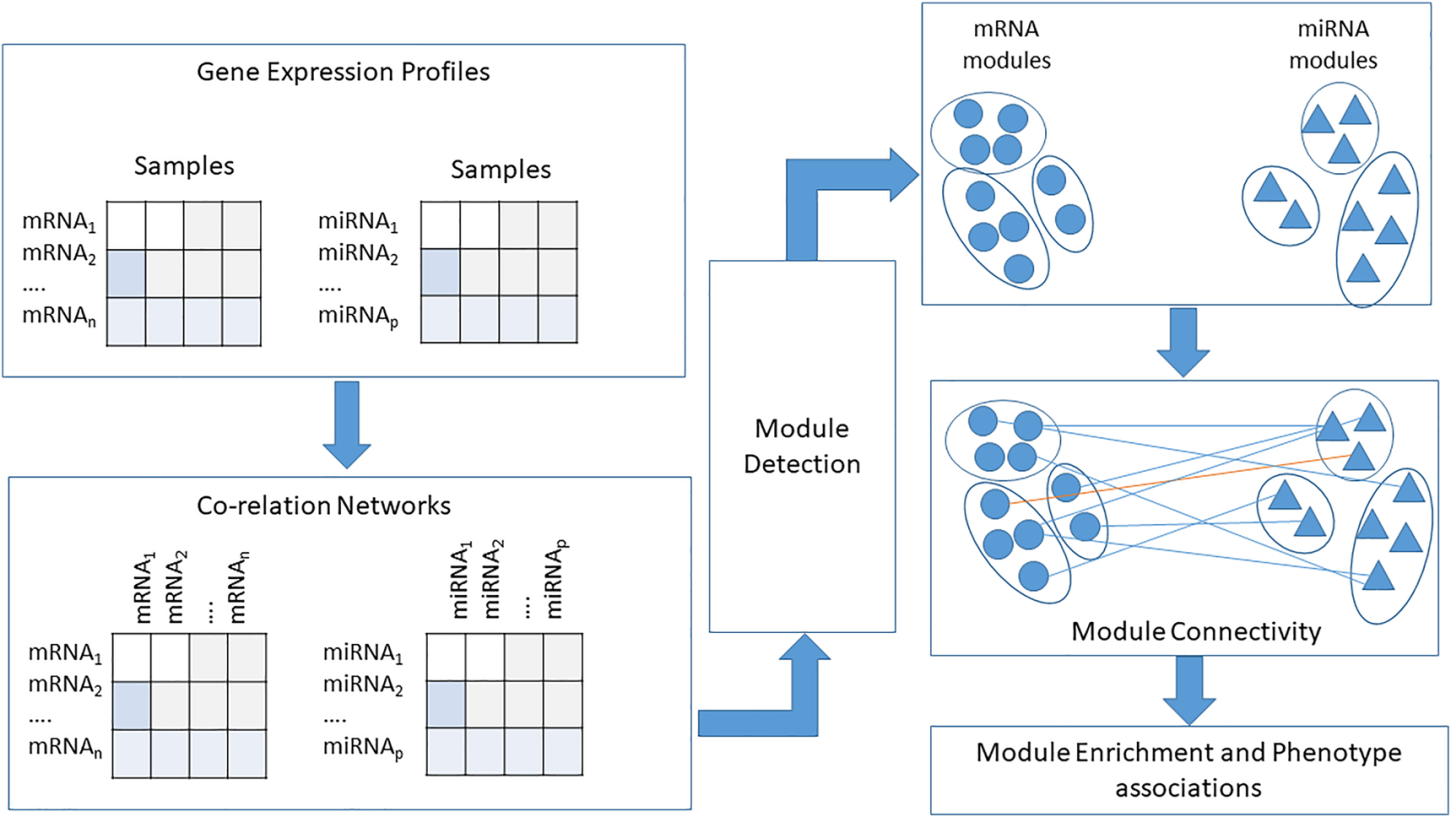
WGCNA based module association workflow.

Therefore, the expression, *a*_*ij*_ = ‖*S*_*ij*_‖^*β*^ is an unsigned network with a scale-free topology represented by an adjacency matrix. The scale-free topology is an inherent property of biological networks. From the weighted adjacency matrix, we obtain a topology overlap matrix (TOM). Using the TOM similarity matrix we get a TOM based dissimilarity matrix d*w*_*ij*_. A hierarchical clustering functionality in WGCNA uses this dissimilarity matrix to identify gene modules. Each module thus identified is assigned a unique color code. We append letters M and G to the color based module names of genes and miRNAs detected by the WGCNA algorithm. Genes/miRNAs, that do not belong to any module are placed into the grey module (i.e., genes/miRNAs in this module are not co-expressed). WGCNA defines a module eigengene (ME), computed as the first principal component that represents the overall expression level of a module. MEs from individual modules (i.e., both gene and miRNA) are used to test the association between phenotypic traits of interest and the identified gene/miRNA modules. We calculate module membership (MM) and gene significance (GS) of all the genes within the detected modules to identify the hubs in each module. Gene significance is calculated as the absolute value of the correlation between a gene and the clinical trait of interest, while module membership of a gene is the correlation between its expression profile and the ME. Genes with high MM and GS values are designated as hubs for that module. A similar procedure is followed for identifying hub miRNAs. A diagrammatic view of this approach is depicted in Figure 2.

## 4 Connecting miRNA–gene modules through interaction network

To establish an interaction network between transcriptional regulatory modules and miRNA regulatory modules we use multiMiR, an R package that provides an interface to 14 external databases housing miRNA-target gene interaction information. The strength of the interaction is assessed depending upon the primary score for target site binding provided by individual databases. We rank the miRNA-target interactions on these score strengths and select top interactions based on either a percentage or a number cutoff. By default, a 20% limit is set which can be customized to obtain a selected set of interactions as per the quality requirements.

## 5 Results

Here, we demonstrate that building co-expression modules of genes and miRNAs using the WGCNA pipeline provide sufficient information about the co-relation between co-expressed gene/miRNA groups and the phenotypic traits. We build relationships to show how a group of genes can jointly regulate and influence the phenotypic outcome of a biological process. Initially, both expression profile datasets are subjected to differential expression analysis. To select statistically significant miRNAs and genes we set the adjusted *p*-value set to 0.05. Applying this filter yields 124 DEMs and 7531 DEGs.

### 5.1 Gene significance and hub gene validation

**Figure 3:**
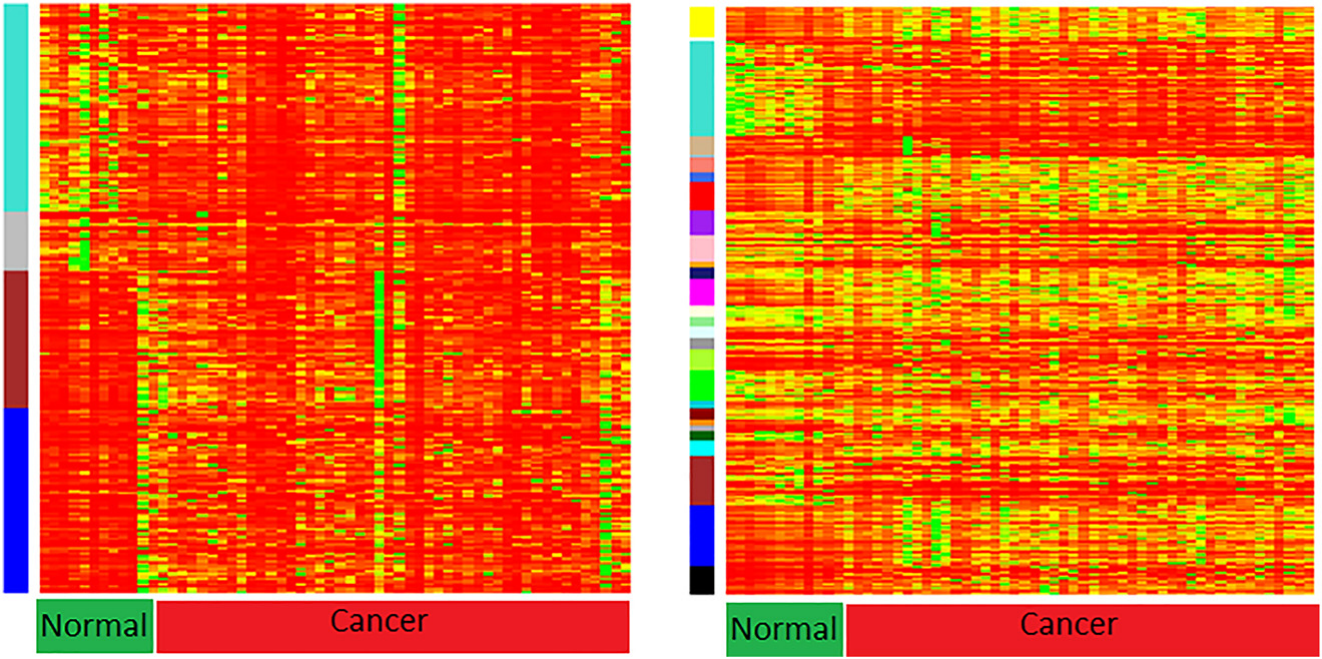
Heatmap showing expression profiles: expression levels in miRNA modules (left) and expression levels in gene modules (right).

**Figure 4:**
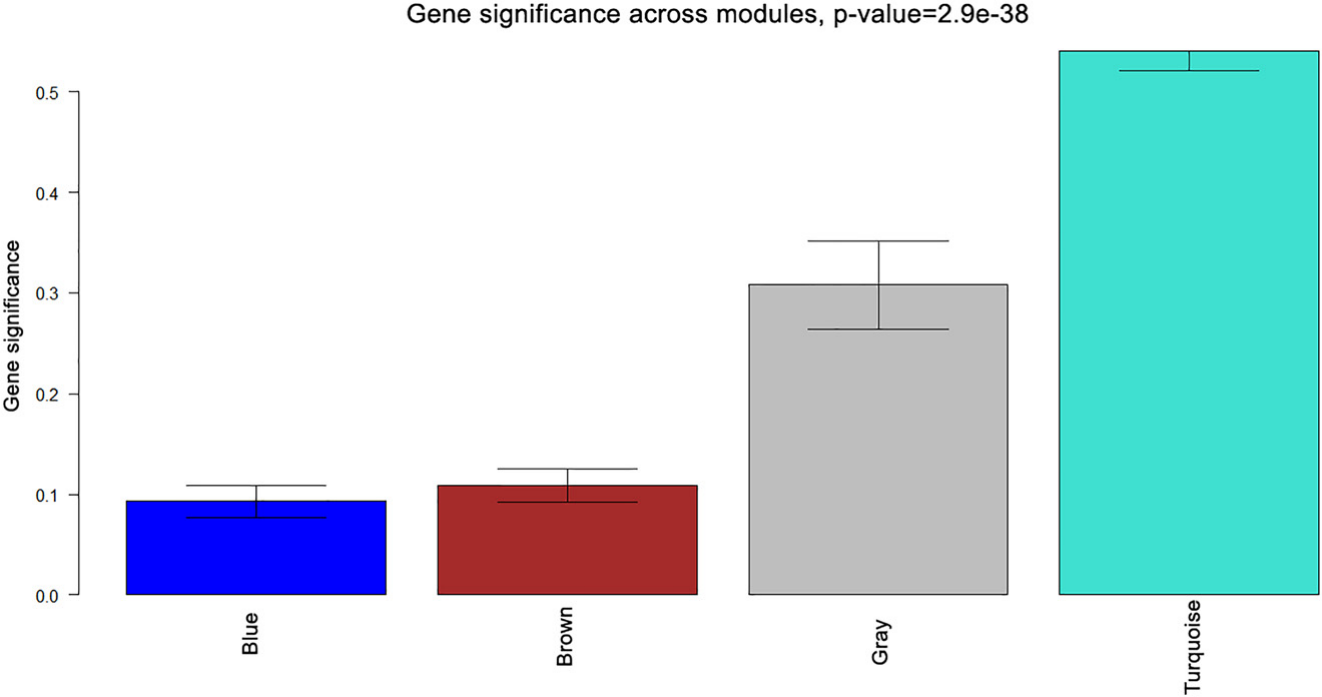
Gene significance of miRNA modules.

**Figure 5:**
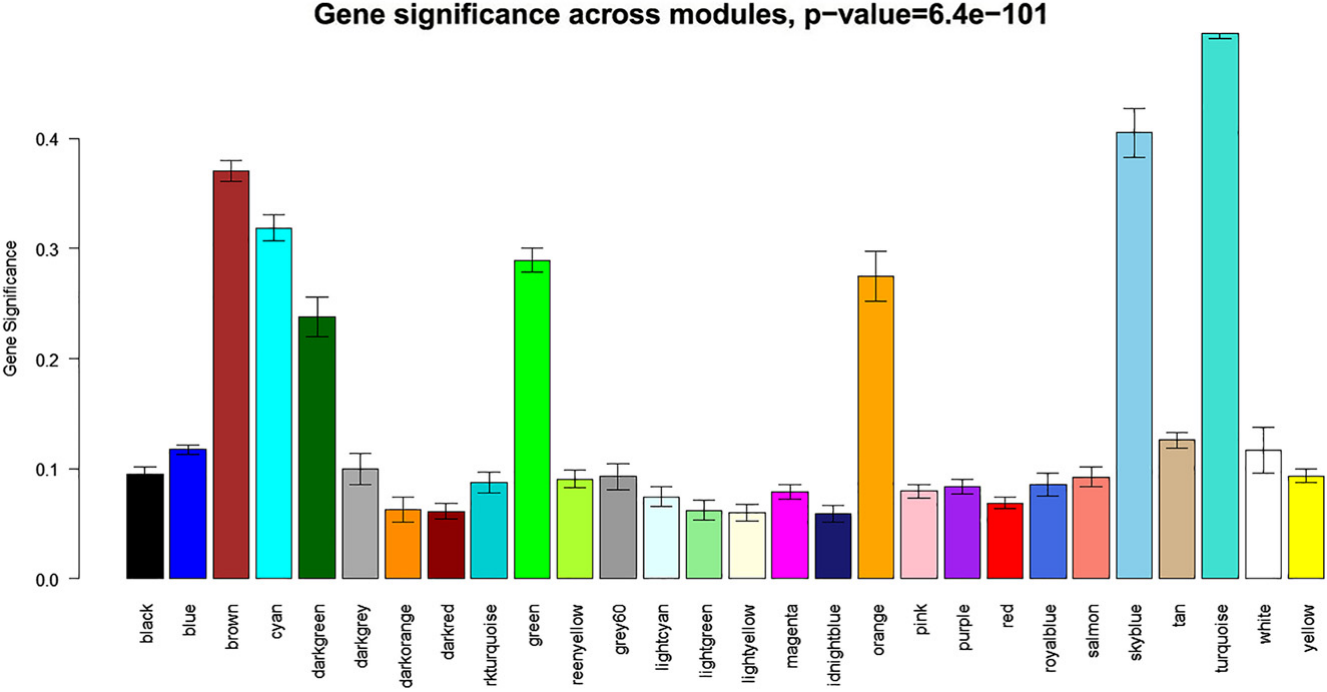
Gene significance of gene modules..

As can be observed from heat maps of both miRNA and gene expression profiles in Figure 3, the turquoise modules seem to cluster most of the miRNAs/genes that show strong expression patterns across cancer samples. To get a clearer picture of the association of various miRNAs/genes to their modules. Also, Figures 4 and 5 plot the gene significance of all the modules of miRNA and gene expression profiles computed as correlation between the expression profiles and the phenotype of interest (e.g., pathological stage and count of lymph nodes).

From the bar plots Figures 4 and 5, it can be observed that grey and turquoise miRNA modules contain miRNAs with higher gene significance. Similarly, brown, dark green, cyan, green, orange, sky blue, and turquoise gene modules contain genes with higher gene significance values corresponding to the clinical traits of interest.

We uploaded the hubs shown in the interaction network to the miRsystem [32] database. A close investigation of the target genes of these miRNA hubs show their enrichment in a number KEGG pathways that control the progression of breast cancer, such as Pathways in Cancer, Axon Guidance, PI3K/AKT/mTOR pathway, neurotrophin signaling pathway, Wnt signaling pathway, and ESR signaling pathway.

### 5.2 Network Analysis

**Figure 6:**
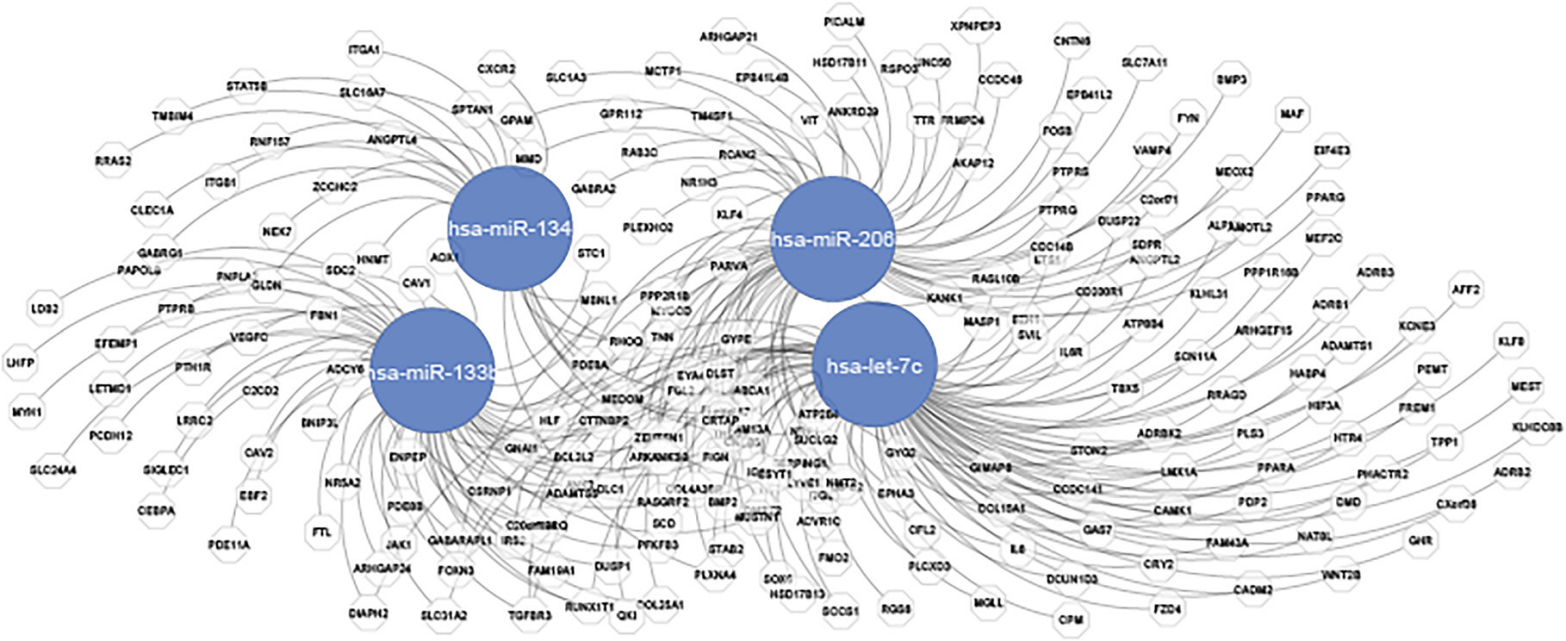
Interaction network between hub miRNAs and genes of turquoise modules.

We also computed the node degree and betweenness centrality topological measures of the turquoise gene and miRNA modules as shown in Table 1. The number of elements involved in integrative regulatory networks that control important aspects of this regulatory network include hubs and genes from turquiose miRNA and gene modules. We apply these important network topological measures to locate central players in the interaction network depicted in Figure 6. The degree of a node also measures how densely it is intertwined with its neighborhood. Such network nodes that possess a very high degree are called hubs [33]. Also, node with high betweenness centrality is considered an influential node in the network.

**Table 1:** Integrative analysis of gene-miRNA regulatory network.

Network node	Degree	Betweenness centrality
hsa-mir-133b	99	0.19
hsa-mir-134	28	0.35
hsa-mir-206	105	0.26
hsa-let-7c	143	0.66

A careful validation analysis of the hubs of this interaction network from the literature reveal that miR-133b and miR-134 are important miRNAs that have been reported to play a tumor suppressor roles in different types of cancers. MiR-133b, which participates in myoblast differentiation and myogenic-related diseases, is commonly recognized as a muscle-specific miRNA [34, 35]. Recent reports demonstrated that miR-133b also plays a crucial role in breast cancer progression and breast metastasis [36]. Another hub-miRNA, mir-134 has also been recently reported to participate in a majority of carcinomas and tumors.

## 6 Discussion & conclusion

In this study, we build weighted co-expression network modules from mRNA and miRNA expression data using the WGCNA R package. We demonstrate module connectivity for both mRNA and miRNA expression profiles by performing the Pearson correlation of module Eigen genes for modules from both mRNA and miRNA data. Heatmaps generated from the expression data depict the correlation between the modules and high/low expression of genes and miRNAs. Also, the gene significance for each module establishes the association between the genes of a module and the phenotypic trait of interest (e.g., cancer in our case). It is observed that genes/miRNAs from turquoise modules have higher gene significance compared to other modules, therefore, we infer an interaction network between hub miRNAs of the turquoise module of miRNA expression data and the genes from the turquoise module derived from gene expression data. An investigation of these hub miRNAs along with some of their targets reveal their enrichment in many signaling pathways associated with cancer progression.

Although module-level regulatory networks inference from expression data help in elucidating the regulatory mechanisms to a greater extent. But incorporating additional knowledge in the form of protein–protein interaction (PPI), gene ontology information and the information from signaling pathways using integrative analysis can further help in exploring intricacies between transcriptional and post-transcription regulatory networks.
